# *SplitTester *: software to identify domains responsible for functional divergence in protein family

**DOI:** 10.1186/1471-2105-6-137

**Published:** 2005-06-01

**Authors:** Xiang Gao, Kent A Vander Velden, Daniel F Voytas, Xun Gu

**Affiliations:** 1 Department of Genetics, Development & Cell Biology, Iowa State University, Ames, Iowa 50011, USA; 2 Bioinformatics and Computational Biology Program, Iowa State University, Ames, Iowa 50011, USA; 3 Pioneer Hi-Bred International, Inc., Johnston, Iowa 50131, USA

## Abstract

**Background:**

Many protein families have undergone functional divergence after gene duplications such that current subgroups of the family carry out overlapping but distinct biological roles. For the protein families with known functional subtypes (a functional split), we developed the software, *SplitTester*, to identify potential regions that are responsible for the observed distinct functional subtypes within the same protein family.

**Results:**

Our software, *SplitTester*, takes a multiple protein sequences alignment as input, generated from protein members of two subgroups with known functional divergence. *SplitTester *was designed to construct the neighbor joining tree (a split cluster) from variable-sized sliding windows across the alignment in a process called *split-clustering*. *SplitTester *identifies the regions, whose split cluster is consistent with the functional split, but may be inconsistent with the phylogeny of the protein family. We hypothesize that at least some number of these identified regions, which are not following a random mutation process, are responsible for the observed functional split. To test our method, we used reverse transcriptase from a group of *Pseudoviridae *retrotransposons: to identify residues specific for diverged primer recognition. Candidate regions were then mapped onto the three dimensional structures of reverse transcriptase. The locations of these amino acids within the enzyme are consistent with their biological roles.

**Conclusion:**

*SplitTester *aims to identify specific domain sequences responsible for functional divergence of subgroups within a protein family. From the analysis of retroelements reverse transcriptase family, we successfully identified the regions splitting this family according to the primer specificity, implying their functions in the specific primer selection.

## Background

Eukaryotic genomes have many genes that fall within well-defined gene or super-gene families [1]. Both orthologuous and paraloguous genes within the same gene family may vary in functions at levels from subtle changes in regulation or catalytic efficiency to substantial evolution of new function. While functional divergence within a protein family is usually determined by changes in a few amino acid residues or domains. Identification of these has traditionally required considerable experimental effort. Developing computational tools for predicting these crucial residues or regions has become important in the field of current functional genomics. Many methods have been proposed, such as ancestral sequence inference [2], positive selection [3], and site-specific rate shifts [4,5]. The new software *SplitTester *reported here is focused on a special type of functional divergence that functionally associated amino acids do not have the same evolutionary relationship as the protein family. The software is designed to identify domains responsible for functional divergence by iteratively comparing split cluster to the functional classification. Identified inconsistence between the functional divergence and the phylogenetic relationship may provide valuable information for gene function prediction.

For illustration, we applied *SplitTester *to the reverse transcriptase family from a group of retrotransposons, *Pseudoviridae*. There are two subgroups of reverse transcriptase, according to the primer utilizations at the initial step of reverse transcription process. One subgroup binds full length tRNA molecule and another one binds tRNA fragment respectively as primer to initiate cDNA synthesis (reviewed in [6] also see [7-9]). Such difference in primer specificity can not be reflected from the inferred phylogenetic relationship of this protein family, that is, they are not monophyletic because of the parallel evolution for functional-related changes during the expansion of this protein family [10]. Thus, one may design a tree-based (clustering) algorithm that can define domains relevant to diverged function in a protein family with known functional subtypes. The software *SplitTester *we developed is to look for the local sequence alignments that display the clustering topology in agreement to a known functional split, using the evolutionary relationship of the gene family as the reference, which can be reconstructed by the conventional methods.

## Implementation

The algorithm implemented in the software *SplitTester *begins with a multiple sequence alignment of a protein family with known functional diversity (functional subgroups), defined here as a 'functional split'. Usually, a functional split is based on a few but unknown diagnostic amino acid residues or regions that are expected to be in accordance with the functional split. If the functional subgroups are not consistent with the phylogenetic tree of the gene family, we may, in retrospect, identify the sequence region that may include amino acid residues crucial for the sought-after function, if the clustering analysis of this region shows the expected functional grouping. In the following we call this idea *the split-clustering *for simplicity.

**Figure 1:**
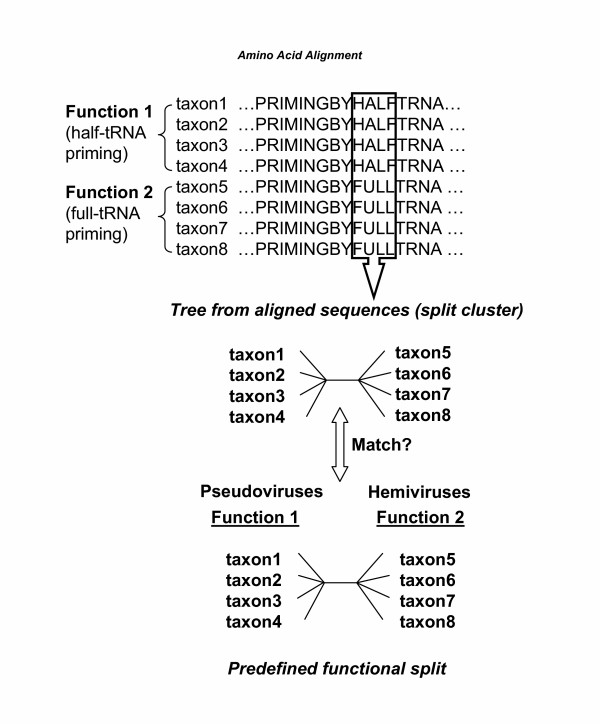
**The algorithm for the tree-based method to identify protein functional domains. **Multiple amino acid sequences alignment is used as an input file. Phylogenetic trees from different windows of the alignment are generated by the neighbor-joining method. For each window, the program determines whether the tree from the local sequence (split cluster) matches a predefined functional split. If the split cluster is consistent with their functional split, the sequence window is a candidate for carrying out that function. The program is iterative and starts with very small windows (i.e. three amino acids), which gradually increase until the window size equals the length of the protein alignment.

Figure 1 illustrates the example for identifying reverse transcriptase amino acid sequences responsible for priming with full or half-tRNAs. Different reverse transcriptases are known to recognize either full length tRNA or tRNA fragment (half-tRNA) as primers, which forms the basis of the known functional split. However, the exact sequence region that is responsible for the primer choice remains unknown yet. The newly-developed method may be helpful to resolve this problem, using the split-clustering approach. That is, by identifying windows of amino acid residues that cluster reverse transcriptases to match the known functional split, one may identify candidate regions that are responsible for primer recognition diversity.

**Figure 2:**
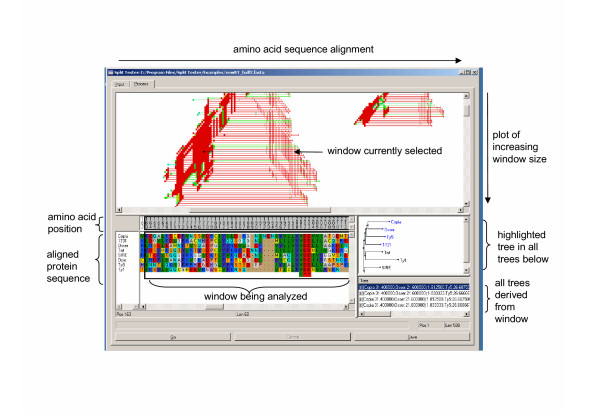
**A snapshot of the Split-Tester software. **A distance matrix was selected to compute phylogenetic relationships of the aligned input sequence data. The regions of the alignment that support the functional split are then plotted in the top window. The X-axis represents the length of the aligned sequences; the Y-axis represents increasing window size. After the computation is complete, the user can select a specific window for analysis by clicking on the left end of colored horizontal bars. The colors indicate the degree of confidence that a given window supports the predefined functional split (red = 100%; yellow = 75%; green = 50%; blue = 25%). The two panels on the lower right show all NJ equivalent trees generated from the selected window. The lower left window shows the actual sequences that support the predefined phylogenetic relationship within the selected window.

We have developed a software package called *SplitTester *(Fig. 2). The input file is the protein sequence alignments, as generated by some conventional methods (e.g. ClustalX) [11]. The users should predefine the functional subtypes, usually based on functional differences of proteins that have been verified by the experimentation. In the current version, the partitioning of sequences is limited to two groups. In the case of multiple functional subtypes, one may start with crude partitions and progressively refining pairs of groups, or by comparing two groups pairwisely. The program uses the neighbor-joining (NJ) method for phylogeny inference or split-clustering [12]. The user can select one of several amino acid substitution distance matrices, including the mutation distance matrix, the hydrophobic distance matrix, PAM10-500 and BLOSUM 30–100 [13,14]. Then, the split-clustering algorithm implemented in *SplitTester *will generate tree-like topologies for each sliding windows along the aligned sequences. The procedure is iterative and starts with very small windows (i.e. three amino acid residues), which slide along the length of the alignment. Window size gradually increases until it reaches the full length of the aligned proteins. All the examined windows are displayed in an plot as part of the output interface. The horizontal axis shows the position of sliding windows in the protein alignment and the vertical axis indicates the length of sliding windows. The tree-topology generated by the split-clustering from each window is compared to the predefined functional split. If it matches, this window is marked as a line on the output plot at the corresponding position and length.

The distance method (e.g. NJ, UPGMA) implemented in most phylogenetic software normally uses the greedy algorithm for efficiency and simplicity, but only tracks a single locally best tree. Consequently, ignoring the alternative solutions can be misleading for phylogeny inference particularly when the sequence length is short [15,16]. To improve the reliability of distance method, there are some efforts to track multiple partial solutions as it progresses [16]. Since split-clustering usually deals with the short sequence length, we modified the conventional algorithm by tracking all possible topologies equally best fitting the data. That is, at each step, the split-clustering searches for the minimal distance pair, creating a list of pairs with equally minimal distance, and then performs the neighbor joining on each pair in order following a depth-first search. Only the resulted unique topology trees are saved for subsequent analyses. In the following we call them NJ-equivalent tree topologies if it is inferred by the NJ algorithm.

If the split-clustering analysis for a given window yields at least one (NJ-equivalent) tree whose topology matches the predefined functional split, we consider the window containing a potential functional signal. In the case of multiple NJ-equivalent trees, as explained above, the strength of the functional signal, or the degree of confidence, is measured by the percentage of NJ-equivalent trees that support the functional split. We used different colors to indicate the signal strength of the window: red means that all (100%) these trees derived from this window split the genes according to the specified function; yellow, green and blue indicate that 75%, 50% and 25% of the trees match the functional split, respectively. A color gradient is used to represent degrees of confidence between the above intervals.

In the output plot, all the lines representing the candidate windows can be selected to display the sequences within this window and the corresponding phylogenetic trees in two separate panels. The panels are updated in real time with the progress of each window tested. While in theory our algorithm could be quite time consuming to run because of the potentially large number of individual NJ operations (time increases quadratically with the length of the alignment), in practice the calculation is very reasonable, because the sequence length is typically small. Our dataset (eight sequences each of 540 amino acid residues) required approximately six minutes for analysis of all possible windows using a Pentium 3 ~ 930 Mhz processor with 512MB RAM and the Microsoft Windows 2000 operating system. In practice, most domains will be identified when window sizes are smaller than 150 amino acids. Therefore, 2–3 minutes of running time is expected to be sufficient for most computers.

*SplitTester *is available as precompiled binary in a distribution package for Microsoft Windows from the following URL: , and . Both a zip file of the installation files and a self-extracting installer are available. Documentation and example files are included in the distribution packages.

## Results and discussion

We applied *SplitTester *to understand functional diversity among retroelement proteins for primer utilization by retroelement reverse transcriptases. Reverse transcriptases of retroviruses (*Retroviridae*), Metaviruses (*Metaviridae*) as well as retrotransposons in the *genus *Pseudovirus (member of *Pseudoviridae *family) use the 3' acceptor stem of the host tRNA as a primer for DNA synthesis. This region pairs with the retroelement RNA template to start DNA synthesis from 3'-OH of the tRNA. Retrotransposons of the *genus *Hemiviruses (member of *Pseudoviridae *family) use a half-tRNA primer, and cDNA synthesis initiates from 3'-OH of nucleotide 40, which resides within the anticodon stem-loop. It is likely that the primer template complexes for the two groups of elements have different structural conformations or properties, and that reverse transcriptases from the different groups have evolved the ability to recognize these differences. The split-clustering implemented by *SplitTester *could explore candidate domains of reverse transcriptase related to primer selection. These identified candidates provide testable hypotheses that could be verified or falsified by the follow-up molecular genetic experimentation.

We focused on members of the *Pseudoviridae*, which include both half-tRNA priming elements (namely the Hemiviruses: Ty5 (U19263), *Osser *(X69552), *1731 *(X07656) and *copia *(M11240)) and elements that use full length tRNAs (namely the Pseudoviruses: Ty1 (M18706), *Opie-2 *(U68408), Tnt1 (X13777) and *SIRE*-1 (AF053008)) [17,18]. The NJ phylogenetic tree from full length sequence alignment did not reflect the divergence of the two functional subtypes (Fig. 4A) [10]. For example, *Osser *and Tnt1 are the only two members of one cluster and the bootstrap value is 74. The genes seem to be clustered according to their hosts, e.g., *1731 *and *copia *from *Drosophila*, Ty5 and Ty1 from *Saccharomyces cerevisiae*, as well as *Osser*, Tnt1, *SIRE-1 *and *Opie-2 *from plants.

**Figure 3:**
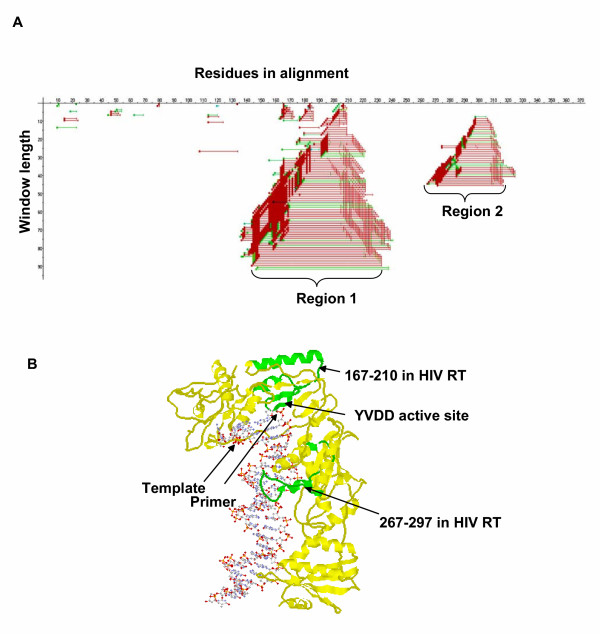
**Functional divergence in reverse transcriptase. **(A) The *SplitTester *output for the reverse transcriptase dataset. Windows supporting the functional split are shown as colored lines in the plot. The X-axis represents the length of the aligned sequences; the Y-axis represents increasing window size (see legend to Fig. 2 for additional detail). (B) The X-ray structure of the HIV reverse transcriptase/primer/template complex (1RTD). The reverse transcriptase protein is represented by the yellow strand. The two green regions are domains identified by *SplitTester*. All residue numbers correspond to HIV sequence positions in 1RTD. Residues 166–215 and 280–311 in the aligned retrotransposon sequences correspond to 167–210 and 267–297 in the HIV 1RTD sequence, respectively.

**Figure 4:**
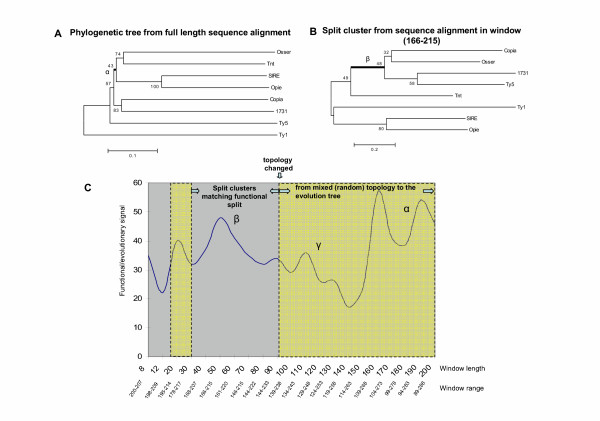
**Phylogenetic relationship from the full length multiple sequence alignment and the predicted region. **(A) NJ phylogeny (MEGA3.0 [32]) from the reverse transcriptase full length sequence alignments clusters the genes from the same host: *Osser*, Tnt1, *SIRE*-1 and *Opie*-2 are from plant host, while *Copia *and *1731 *are from *Drosophila*. Ty1 and Ty5 are from *Saccharomyces cerevisiae*. (B) Split cluster from the window length of 50 aa (position 166–215) in the predicted region 1 supports the functional subtype split. (C) Functional signal (measured by the bootstrap of node β in panel B), as well as the evolutionary background (measured by the bootstrap of node α in panel A), plotted against the window size. In the window length less than 90, the split-clustering supports the functional subtypes split and the bootstrap value reach the peak in window with length around 50 aa. A mixed topology is detected when window length is longer than 90 aa, measured by the bootstrap (γ) between two major subtrees. When more amino acid sites are included, the bootstrapping value converges to the node α in panel A.

Two signal regions were identified by *SplitTester *when the mutation distance matrix was used to compute the cost of amino acid substitution and when gaps were considered as potentially informative characters. In the aligned amino acid sequence, the maximum windows showing functional split in each non-overlapping region cover positions 144–239 (region 1) and 269–314 (region 2) (Fig. 3A). Using the hydrophobicity matrix, *SplitTester *also identified these regions, as well as an extra region at the N-terminus (amino acids 1–29, region 3). Each of signal regions above is derived from the multiple overlapping signal windows with gradually increased window length. We chose 50 aa window (166–215) in region 1 and 32 aa window (280–311) in region 2. Both windows are close to the median window length with demonstrating high bootstrapping values (Fig. 4C). In general, one may use the median sized window for representing, because small windows are statistically unstable while a too broad window will demolish the functional signal of the window.

We located the two windows identified by both matrices within the HIV reverse transcriptase sequence, based on the published protein sequence alignments of the reverse transcriptase family by Xiong and Eickbush [19], and mapped onto the crystal structure 1RTD [20] (Fig. 3B). HIV reverse transcriptase p66 has a 'right hand' structure. The 50 aa window (166–215) in region 1 from the alignment correspond to the "palm" of the protein (residues 167–210 in HIV reverse transcriptase) that encompasses the polymerase active site wherein nucleotides are added to the 3' end of the primer. The 32 aa window (280–311) in region 2 corresponds to one α-helix in the 'thumb' region of HIV reverse transcriptase (residues 267–297), which directly contact the primer/template complex, as determined by cross-linking experiments [21,22].

The region of β-sheets between the two identified domains (240–268 in the retroelement reverse transcriptase alignment) is called the primer grip and cross-links to the 3'-OH group of the HIV primer, tRNA^lys ^[23,24]. We would speculate that primer grip is related to the primer binding. However, this region is not identified as determinant of primer specificity, indicating that the ability of the primer grip to interact with the 3'-OH of the primer will be a common feature of both full-and half-tRNA priming retroelements. The two regions identified by *SplitTester *surrounding the primer grip may play a role in distinguishing primer conformation or length. The results of *SplitTester*, therefore, can be well explained from the reverse transcriptase crystal structure and supported by experimental data. To further validate our findings, we randomly partitioned the gene members into two groups and reran the program. The candidate functional regions were not identified using any of the partitions (data not shown). We therefore conclude that the identified residues may separate the functional subtypes specifically.

The strength of functional signal vs. the evolutionary background on the multiple windows of different length (from 8–200) in the signal region 1 can be well illustrated by the bootstrapping values (Fig. 4C). The right splitting of the two functional groups in this region (29-90aa), as predicted by *SplitTester*, showing a (local) maxima at the window length of 50 residues. Interestingly, when the window size is greater than 90, the topology by the split-clustering is a random mixture between functional split and evolutionary background; the bootstrapping value is for the deepest two subtrees. Rather, with the increase of the window length toward the window of full length sequence, the topology converges to the evolutionary tree as shown in Fig. 4A. Therefore, the region identified by *SplitTester *is intuitively valid even though although the bootstrapping (re-sampling) value is not very high. We also observed the very similar pattern for region 2; the highest bootstrap value in window with size of 32 aa around the median length within the range of 8 to 46.

Because the *SplitTester *employs a heuristic algorithm to decipher weak functional signal from the high level of evolutionary background, its power would be limited by several factors. If there are only a few amino acid residues related to a given functional split, or these residues are located in a broad sequence region, the statistical power for detection is low, as we expected. One improvement for *SplitTester *is to divide the proteins into several small regions, for instance, based on the protein structure, or motifs. Then one can implement some algorithms to integrate weak signals from these non-overlapping small regions, which otherwise are indistinguishable from the background.

There are several other factors that may affect the performance of *SplitTester*. The first one is the choice of substitution matrices of proteins. We have provided several options for the matrices. The user can select one of them based on their purpose. In general, BLUSOM matrices reflect the overall evolution history and hydrophobicity matrix can reflect more on chemistry properties of amino acids. Second, the inference power of this method might increase with the difference between the functional splitting and phylogenetic tree because the signals are more likely being distinguished from the background. Thus, increasing the sample size, for instance, sequencing more genes from different species is certainly helpful. Nevertheless, the example of reverse transcriptases we presented here has indicated that one may obtain valuable functional information even the difference between functional splitting and phylogenetic tree is weak as indicated by the bootstrap value.

There have been many methods developed to analyze sequences involved in functional divergence between protein subtypes (e.g., [25,26,4]). Most of them are focused on identifying specific residues contributing to the functional differences of subtypes along the phylogeny. Hence, the prediction accuracy may rely on the quality of multi-alignments, the accuracy of phylogenetic inference, or the sufficient number of sequences. Since the *SplitTester *focus on a particular sequence region that supports the subtype functional split, the predicted results seem not to be strongly affected if a few positions are misaligned. We validated this claim by deleting the position 166 in the multiple sequence alignment, which contain conserved F in Pseudovirus subfamily and A, V, E, T in each gene of Hemevirus subfamily respectively. This position was selected because it can be easily identified manually as one of three "seeds" of the growing signal region (Fig. 3A). Fortunately, *SplitTester *still identified the almost identical region 1 as before only in overlapped shorter windows (145–221) and (167–238) (data not shown). Similar results were obtained when we deleted each of other two "seeds" at position 184 (conserved K only in Pseudovirus subfamily) and 207 (conserved A only in Hemivirus subfamily).

## Conclusion

*SplitTester *can explore regions potentially responsible for functional divergence of proteins. The best scope of this software is to study the dataset with different functional clustering and phylogenetic tree. As shown by the case of reverse transcriptase, function-related signals may emerge when the functional split is inconsistent with the phylogenetic relationship of the protein family. Even if the functional clustering is consistent with its phylogeny, *SplitTester *may also provide some useful information for amino acid residues important for functional divergence, e.g., the conserved Myb gene family. In spite that the phylogeny is the same as the known functional split of Myb genes, *SplitTester *still successfully identified 11 candidate residues that differentiate the two-and three-repeat Myb proteins, the major functional split of Myb gene family [27], because the sequence window including these residues shows a stronger functional signal. Indeed, these identified candidate residues are well supported by their locations on the NMR structure of the mouse c-Myb DNA binding domain 1MSF [28]. Moreover, these residues are called by type-II functional divergence by [29], which can also be predicted by the "Evolution Trace" method [30]. See additional files 1 and 2 for the detail. Finally, we mention that, after combining *SplitTester *with other complementary methods, such as the method of [25], "Evolution Trace" method [30], "Phylogenetic Inference" by Sjolander [31], Diverge [5], we can develop a powerful analysis pipeline for predicting functional divergence from sequence domains to amino acid residues.

In summary, we developed *SplitTeste*r – a tool for exploring the functional domains in protein family. *SplitTester *focus on a specific type of functional divergence: the functional split is different from the evolutionary relationship. Using the split-clustering algorithm, *SplitTester *scans all the possible local regions of protein sequence alignment to identify the domain that provide the same clustering topology as the functional split. In the analysis of retroelements reverse transcriptase family, we identified the regions splitting this family according to the primer specificity, implying function in the primer selection. The functional role can be well explained after we map the identified domain onto the structure of the reverse transcriptase protein.

## Availability and requirements

• project name: SplitTester

• Project home page:



• Operating system(s): windows 2000 and XP

• Programming language: C

## Authors' contributions

XGao designed this project, analyzed the results and prepared the manuscript. KVV implemented the software. XGu and DFV supervised the project and helped to prepare the manuscript. All authors read and approved the final manuscript.

## Supplementary Material

Additional File 1 Display the result of identified residues in MyB protein. Click here for file

Additional File 2 Descript the detail of Myb protein family and the predicted key residues for functional divergence from *SplitTester*. Click here for file

## References

[B1] Tatusov RL, Koonin EV, Lipman DJ (1997). A genomic perspective on protein families. Science.

[B2] Golding GB, Dean AM (1998). The structural basis of molecular adaptation. Mol Biol Evol.

[B3] Yang Z, Bielawski JP (2000). Statistical methods for detecting molecular adaptation. Trends in Ecology and Evolution.

[B4] Gu X (1999). Statistical methods for testing functional divergence after gene duplication. Mol Biol Evol.

[B5] Gu X, Vander Velden K (2002). DIVERGE: phylogeny-based analysis for functional-structural divergence of a protein family. Bioinformatics.

[B6] Leis J, Aiyar A, Cobrinik D, Goff S and Skalka A (1993). Regulation of initiation of reverse transcription of retroviruses. Reverse transcriptase.

[B7] Kikuchi Y, Ando Y, Shiba T (1986). Unusual priming mechanism of RNA-directed DNA synthesis in copia retrovirus-like particles of Drosophila. Nature.

[B8] Chapman KB, Bystrom AS, Boeke JD (1992). Initiator methionine tRNA is essential for Ty1 transposition in yeast. Proc Natl Acad Sci U S A.

[B9] Ke N, Gao X, Keeney JB, Boeke JD, Voytas DF (1999). The yeast retrotransposon Ty5 uses the anticodon stem-loop of the initiator methionine tRNA as a primer for reverse transcription. Rna.

[B10] Boeke JD, Eickbush T, Sandmeyer SB, Voytas DF, van Regenmortel MHV, Fauquet CM, Bishop DHL, Carsten EB, Estes MK, Lemon SM, Maniloff J, Mayo MA, McGeoch DJ, Pringle CR and Wickner RB (2000). Pseudoviridae. Virus Taxonomy: Seventh Report of the International Committee on Taxonomy of Viruses.

[B11] Jeanmougin F, Thompson JD, Gouy M, Higgins DG, Gibson TJ (1998). Multiple sequence alignment with Clustal X. Trends Biochem Sci.

[B12] Saitou N, Nei M (1987). The neighbor-joining method: a new method for reconstructing phylogenetic trees. Mol Biol Evol.

[B13] Henikoff S, Henikoff JG (1992). Amino acid substitution matrices from protein blocks. Proc Natl Acad Sci U S A.

[B14] Levitt M (1976). A simplified representation of protein conformations for rapid simulation of protein folding. J Mol Biol.

[B15] Maddison PJ (1991). Overlap syndromes and mixed connective tissue disease. Curr Opin Rheumatol.

[B16] Pearson WR, Robins G, Zhang T (1999). Generalized neighbor-joining: more reliable phylogenetic tree reconstruction. Mol Biol Evol.

[B17] Fourcade-Peronnet F, d'Auriol L, Becker J, Galibert F, Best-Belpomme M (1988). Primary structure and functional organization of Drosophila 1731 retrotransposon. Nucleic Acids Res.

[B18] Voytas DF, Boeke JD (1992). Yeast retrotransposon revealed. Nature.

[B19] Xiong Y, Eickbush TH (1990). Origin and evolution of retroelements based upon their reverse transcriptase sequences. Embo J.

[B20] Huang H, Chopra R, Verdine GL, Harrison SC (1998). Structure of a covalently trapped catalytic complex of HIV-1 reverse transcriptase: implications for drug resistance. Science.

[B21] Jacobo-Molina A, Ding J, Nanni RG, Clark ADJ, Lu X, Tantillo C, Williams RL, Kamer G, Ferris AL, Clark P (1993). Crystal structure of human immunodeficiency virus type 1 reverse transcriptase complexed with double-stranded DNA at 3.0 A resolution shows bent DNA. Proc Natl Acad Sci U S A.

[B22] Peletskaya EN, Boyer PL, Kogon AA, Clark P, Kroth H, Sayer JM, Jerina DM, Hughes SH (2001). Cross-linking of the fingers subdomain of human immunodeficiency virus type 1 reverse transcriptase to template-primer. J Virol.

[B23] Arnold E, Ding J, Hughes SH, Hostomsky Z (1995). Structures of DNA and RNA polymerases and their interactions with nucleic acid substrates. Curr Opin Struct Biol.

[B24] Wohrl BM, Tantillo C, Arnold E, Le Grice SF (1995). An expanded model of replicating human immunodeficiency virus reverse transcriptase. Biochemistry.

[B25] Casari G, Sander C, Valencia A (1995). A method to predict functional residues in proteins. Nat Struct Biol.

[B26] Hannenhalli SS, Russell RB (2000). Analysis and prediction of functional sub-types from protein sequence alignments. J Mol Biol.

[B27] Jiang C, Gu J, Chopra S, Gu X, Peterson T (2004). Ordered origin of the typical two- and three-repeat Myb genes. Gene.

[B28] Ogata K, Morikawa S, Nakamura H, Sekikawa A, Inoue T, Kanai H, Sarai A, Ishii S, Nishimura Y (1994). Solution structure of a specific DNA complex of the Myb DNA-binding domain with cooperative recognition helices. Cell.

[B29] Gu X (2003). Functional divergence in protein (family) sequence evolution. Genetica.

[B30] Lichtarge O, Bourne HR, Cohen FE (1996). An evolutionary trace method defines binding surfaces common to protein families. J Mol Biol.

[B31] Sjolander K (1998). Phylogenetic inference in protein superfamilies: analysis of SH2 domains. Proc Int Conf Intell Syst Mol Biol.

