# Supplementary figures and images for: SplitTester : software to identify domains responsible for functional divergence in protein family
Source: BMC Bioinformatics. 2005 Jun 1;6:137. doi: 10.1186/1471-2105-6-137 (PMC1181622; doi:10.1186/1471-2105-6-137)

## Slide 1
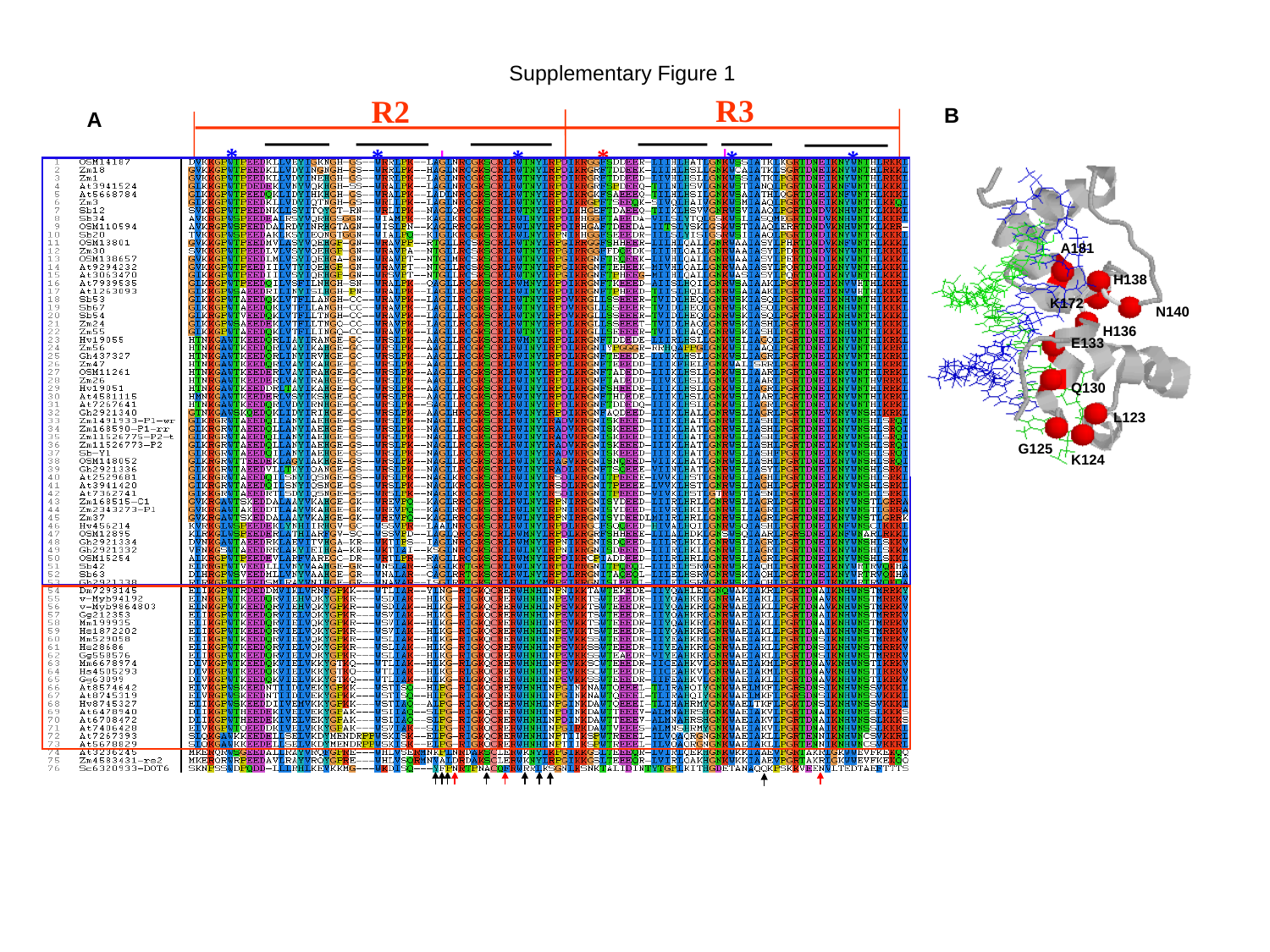

Supplementary Figure 1
R2
R3
|
*
*
*
|
*
*
*
B
A
A181
H138
K172
N140
H136
E133
Q130
L123
G125
K124

Supplement: Additional File 1 — Display the result of identified residues in MyB protein. [file 1471-2105-6-137-S1.ppt]
