# Supplementary material for: SplitTester : software to identify domains responsible for functional divergence in protein family
Source: BMC Bioinformatics. 2005 Jun 1;6:137. doi: 10.1186/1471-2105-6-137 (PMC1181622; doi:10.1186/1471-2105-6-137)
Supplement: Additional File 2 — Descript the detail of Myb protein family and the predicted key residues for functional divergence from SplitTester. [file 1471-2105-6-137-S2.doc]

Supplementary Figure 1 --

We used 53 three-repeat Myb proteins and 20 two-repeat Myb proteins from plants, animals, fungi and viruses. *SplitTester* running result indicates that the functional divergence is consistent with the evolutionary history of these proteins, because the size of the signal regions increase with the window size until they cover the full length of proteins. *SplitTester* found 11 residues that differentiate the two- and three-repeat Myb proteins (supplementary figure 1A). All of these sites were mapped on the NMR structure of the mouse c-Myb DNA binding domain, a three-repeat protein, except for Leu126, which is deleted in this subtype (supplementary figure 1B) [28]. Most sites identified by *SplitTester* face the major groove of DNA, implying that the difference between the two types of Myb domains is related to DNA binding activity. Furthermore, most sites are located in the third α-helix of each repeat unit. This is consistent with experimental results indicating that the third α-helix of each repeat plays a role in DNA recognition. Among the 11 sites identified, three have been experimentally related to DNA binding: an insertion at Leu126 or a substitution at Ala180 compromise DNA interactions in the three-repeat Myb proteins; in the two-repeat Myb proteins, a mutation in Leu55 (which corresponds to Glu132 in the three-repeat Myb proteins) similarly affects interactions with DNA. In summary, *SplitTester* was effective in identifying residues that likely determine functional diversity among this conserved protein family, whose functional divergency are very likely in agreement with its phylogenetic relationship.
